# Development of Human-Like scFv-Fc Neutralizing Botulinum Neurotoxin E

**DOI:** 10.1371/journal.pone.0139905

**Published:** 2015-10-06

**Authors:** Sebastian Miethe, Christine Rasetti-Escargueil, Arnaud Avril, Yvonne Liu, Siham Chahboun, Hannu Korkeala, Christelle Mazuet, Michel-Robert Popoff, Thibaut Pelat, Philippe Thullier, Dorothea Sesardic, Michael Hust

**Affiliations:** 1 Technische Universität Braunschweig, Institut für Biochemie, Biotechnologie und Bioinformatik, Abteilung Biotechnologie, Spielmannstr. 7, 38106 Braunschweig, Germany; 2 Division of Bacteriology, National Institute for Biological Standards and Control (NIBSC), a centre of Medicines and Healthcare products Regulatory Agency, Blanche Lane, South Mimms, Potters Bar, Hertfordshire EN6 3QG, United Kingdom; 3 Institut de Recherche Biomédicale des Armées (IRBA-CRSSA), Département de Microbiologie, Unité de biotechnologie des anticorps et des toxines, 24 avenue des Maquis du Grésivaudan, B.P. 87, 38702 La Tronche Cedex, France; 4 University of Helsinki, Faculty of Veterinary Medicine, Centre of Excellence in Microbial Food Safety Research, Department of Food Hygiene and Environmental Health, P.O. Box 66 (Agnes Sjöbergin katu 2), 00014 Helsinki University, Helsinki, Finland; 5 Unité des Bactéries anaérobies et Toxines, Institut Pasteur, 25 avenue du Docteur Roux, 75015, Paris, France; Naval Research Laboratory, UNITED STATES

## Abstract

**Background:**

Botulinum neurotoxins (BoNTs) are considered to be the most toxic substances known on earth and are responsible for human botulism, a life-threatening disease characterized by flaccid muscle paralysis that occurs naturally by food-poisoning or colonization of the gastrointestinal tract by BoNT-producing clostridia. BoNTs have been classified as category A agent by the Centers of Disease Control and Prevention (CDC) and are listed among the six agents with the highest risk to be used as bioweapons. Neutralizing antibodies are required for the development of effective anti-botulism therapies to deal with the potential risk of exposure.

**Results:**

In this study, a macaque (*Macaca fascicularis*) was immunized with recombinant light chain of BoNT/E3 and an immune phage display library was constructed. After a multi-step panning, several antibody fragments (scFv, single chain fragment variable) with nanomolar affinities were isolated, that inhibited the endopeptidase activity of pure BoNT/E3 *in vitro* by targeting its light chain. Furthermore, three scFv were confirmed to neutralize BoNT/E3 induced paralysis in an *ex vivo* mouse phrenic nerve-hemidiaphragm assay. The most effective neutralization (20LD_50_/mL, BoNT/E3) was observed with scFv ELC18, with a minimum neutralizing concentration at 0.3 nM. Furthermore, ELC18 was highly effective *in vivo* when administered as an scFv-Fc construct. Complete protection of 1LD_50_ BoNT/E3 was observed with 1.6 ng/dose in the mouse flaccid paralysis assay.

**Conclusion:**

These scFv-Fcs antibodies are the first recombinant antibodies neutralizing BoNT/E by targeting its light chain. The human-like nature of the isolated antibodies is predicting a good tolerance for further clinical development.

## Introduction

Botulism is a potentially life-threatening disease associated with foodborne poisoning caused by intoxication with botulinum neurotoxins (BoNTs) that are secreted by *Clostridium botulinum* and certain other *Clostridium spp*. or by colonization of the gastrointestinal tract by BoNT-producing clostridia. It is characterized by flaccid and life-threatening muscle paralysis that requires long term treatment in intensive care unit [1,2]. To date, 7 serologically distinct serotypes (designated A to G) of BoNT are known. Four BoNT serotypes (A, B, E, rarely F) are responsible for most cases of human botulism [3]. In the past, several cases of botulism caused by BoNT/A, B and E have been reported worldwide [4–8]. BoNT/F causes only 1% of food poisoning-related cases of botulism, including adult toxin coinfections [9]. Due to their high toxicity, BoNTs are classified as category A agents by the Centers for Disease Control and Prevention (CDC) and as a group are among the six agents with the highest risk of potential use as bioweapons [10,11]. The Soviet Union and Iraq were suspected of weaponizing BoNTs and the Japanese cult AUM Shinrikyo attempted to use BoNT for bioterrorism [12,10,13]. Furthermore, the risk of deliberate contamination of the food chain by BoNTs has been highlighted by several incidents [14].

*Clostridium spp*. producing botulinum neurotoxin E (BoNT/E) are found in aquatic sediments and intoxications are reported mainly in North European, American and Asian regions where fish are smoked or frequently eaten without cooking [15]. BoNT/E-related intoxications are more scarce than those related to BoNT/A and BoNT/B, but the median lethal dose 50 (LD_50_) of BoNT/E is estimated to be as low as that of BoNT/A, equal to 1.1 ng/kg in mice and monkeys (by intravenous, subcutaneous and intraperitoneal route) [16]. To date, twelve BoNT/E subtypes (E1 to E12) have been identified based on sequence analysis [17].

All BoNT serotypes are synthesized as a 150 kDa single-chain progenitor toxin which is subsequently activated by a clostridial protease to generate a disulfide bond-linked structure containing a 50 kDa light chain and a 100 kDa heavy chain. The heavy chain contains two functional domains (Hc and Hn) that are required for toxin uptake into nerve cells by receptor-mediated endocytosis and for the translocation of the light chain across the membrane into the neuronal cytosol. In a final step, the catalytic domain of the light chain (a zinc endopeptidase), cleaves the SNARE complex proteins (soluble N-ethylmaleimide-sensitive factor attachment protein receptor) which are involved in the fusion of synaptic vesicles with the presynaptic membrane and inhibits neurotransmitter release, thereby causing flaccid paralysis, which requires intensive hospitalization [18]. Contrary to other BoNT serotypes, BoNT/E, produced by group II *C*. *botulinum* strains, is secreted by clostridia as a unique inactive chain that requires activation by host proteases for toxicity. This process, called nicking, is associated with a 100-fold increase in toxicity [19,20]. After BoNT/E binds to specific receptors on the surface of neurons and following endocytosis and translocation of the light chain into the cytosol, the toxin acts on peripheral cholinergic nerve endings, where it cleaves SNAP–25 (synaptosomal-associated protein 25 kDa) through its zinc metalloprotease activity, targeting specifically between residues 180 and 181, resulting in the inhibition of acetylcholine release at the neuromuscular junction by vesicle exocytosis [21,22].

BoNTs are also used as therapeutics for a multitude of disorders including glandular hypersecretion, skeletal or smooth muscle hyperactivity and chronic pain-associated involuntary muscle conditions [23,24]. Due to the increasing medical uses of BoNTs, widespread vaccination against botulism would prevent from the benefit of these wide therapeutic applications. The current approach for treatment of botulism is based on passive immunization with an equine antitoxin sera consisting of Fab and/or F(ab')_2_ preparations [25] or in case of infant botulism with human anti-botulism immunoglobulins, such as BabyBig^®^ [26]. Unfortunately, the quantity of the human serum stock is limited [27] and the equine sera may induce serious adverse effects, including serum sickness and hypersensitivity [28]. The efficiency of these antitoxin treatments is removal of the toxin from the bloodstream before it can be internalized into neurons and induce a lethal flaccid paralysis. However, these antitoxins are no longer effective once the toxin is taken up into the neurons which happens faster than the identification of a botulism case since patients develop symptoms more than 3 days after intoxication. The current situation supports the need for new human-like or human antibody preparations that are highly effective and better tolerated than equine antibodies. However, because BoNT serotypes differ by up to 70% in their amino acid sequence, it is necessary to neutralize each serotype with specific antibodies [29].

To develop neutralizing antibodies against BoNT/E, the selection of high affinity antibodies using non-human primate immune libraries is a promising strategy. Due to the phylogenetic proximity between non-human primates (NPHs), such as macaques (*Macaca fascicularis*), and humans, the use of NHPs makes it possible to isolate human-like antibody fragments for therapeutic applications [30]. This strategy has been successfully used for the isolation of several antibody fragments with nanomolar or picomolar affinities against different antigens of clinical interest, such as Crf2, a surface antigen of *Aspergillus fumigatus [31]*, viruses like Western equine encephalitis virus (WEEV) and Venezuelan equine encephalitis virus (VEEV) [32,33] and several toxins, such as tetanus toxin [34], ricin [35] and the two sub-units of anthrax lethal toxin [36,37]. In a previous study we isolated several recombinant antibodies targeting the light chain of BoNT/A, using a macaque immune library, which neutralize the toxicity of BoNT/A in an *ex vivo* mouse phrenic nerve diaphragm assay [38]. In recently published studies, the epitopes of the antibodies generated against BoNT/E were located in the heavy chain. Previously, seventeen monoclonal antibodies were generated by immunization of BALB/c mice with type E toxoid, which bound to the heavy chain of BoNT/E [39]. Furthermore, an antibody, scFv 4E17, was isolated from human volunteers immunized with botulinum pentavalent vaccine, which binds to an epitope located at the N-terminus of the heavy chain [40]. Meng et al. described a human monoclonal antibody directed against an epitope located on the light chain of BoNT/E [41]. However, to our knowledge, no recombinant human-like antibody that neutralizes BoNT/E *in vivo* by targeting the light chain (BoNT/E-L) has been reported to date.

Our strategy for the isolation of BoNT/E-L specific antibody fragments was based on the immunization of a macaque with recombinant BoNT/E3-L to generate an immune library, potentially spanning most of the epitopes on the light chain of BoNT/E with high-affinity antibodies, rather than selection of antibodies directed against non-relevant antigens. In this study we describe the isolation and characterization of several scFv from a non-human antibody gene library by phage display, focusing on their inhibition of the protease activity of BoNT/E-L *in vitro*, the neutralization properties in the mouse *ex vivo* phrenic nerve model as scFv and scFv-Fc and *in vivo* protection in the mouse flaccid paralysis assay.

## Materials and Methods

### Ethical statement and animal care

All animal studies described involving the use on non-human primates received specific approval from the Institut de Recherche Biomédicale des Armées Ethics committee (Comité d’éthique de l’Institut de Recherche Biomédicale du Service de Santé des Armées) under authorization no. 2008/03.0 and were performed in accordance with all relevant French laws and ethical guidelines, including, in particular (1) “partie règlementaire du livre II du code rural (Titre I, chapitre IV, section 5, sous-section 3: expérimentation sur l’animal),” (2) “décret 87–848 du 19-10/1987 relatif aux expériences pratiquées sur les animaux vertébrés modifié par le décret 2001/464 du 29/05/2001,” (2) “arrêté du 29 octobre 1990 relatif aux conditions de l’expérimentation animale pour le Ministère de la Défense,” and (4) “instruction 844/DEF/DCSSA/AST/VET du 9 avril 1991 relative aux conditions de réalisation de l’expérimentation animale.” Animal care procedures complied with the regulations detailed under the Animal Welfare Act [42] and in the Guide for the Care and Use of Laboratory Animals [43]. Animals were kept at a constant temperature (22°C ± 2°C) and relative humidity (50%), with 12 h of artificial light per day. They were housed in individual cages (6 per room), each of which contained a perch. Animals were fed twice daily, once with dried food and once with fresh fruits and vegetables, and water was provided at the same time. Food intake and general behavior were observed by the animal technicians during feeding times and veterinary surgeons were available for consultation if necessary. Veterinary surgeons also performed regular visits to each NHP-room twice weekly. The environmental enrichment program for the non-human primates involved games with animal care staff and access to approved toys. The well-being of the animals was monitored by the attending veterinary surgeon. Animals were anesthetized before the collection of blood or bone marrow by an intramuscular injection of 10 mg/kg ketamine (Imalgene^®^, Merial). If the animal technicians suspected that the animal was in pain, on the basis of their observations of animal behavior, analgesics were subsequently administered, through a single intramuscular injection of 5 mg/kg flunixine (Finadyne^®^, Schering Plough) in the days after interventions. None of the non-human primates died during this study.

For the *ex vivo* phrenic nerve-hemidiaphragm assays male in-bred BALB/c mice supplied by Harlan (UK) were used. The mice were killed by cervical dislocation prior to test. Death was then confirmed by severing the jugular blood vessel to ensure permanent cessation of the circulation. For the *in vivo* paralysis assay female, outbred, strain MF1 mice were used, supplied by Harlan (UK). The procedure was a refinement of the lethality (LD50) test, as only sub-lethal doses of toxin were injected into animals and no animal died during the process. The *in vivo* paralysis assay was performed at NIBSC by approved procedure covered by the UK Home Office project license (PPL # 80/2634, holder Dr D Sesardic) for Research on Bacterial Products used in Medicine. All such experiments complied with the UK Home Office regulations for the use of animals in research and testing under the Animals (Scientific Procedures) Act 1986 (ASPA) and revised European Directive 2010/63/EU on the protection of animals.

All experiments on animals at NIBSC (UK) were approved by the local animal research oversight committee AWERB (Animal Welfare and Ethics Review Body, which meets monthly to review procedures.

### Production of Botulinum Neurotoxin E light chain for animal immunization

DNA encoding the BoNT/E light chain (amino acids 1–420) was PCR amplified from *C*. *botulinum* E3 strain P34 with primers adding *Bam*HI and *Sal*I restriction sites at the 5' and 3' ends, respectively. The DNA fragment was cloned into pET28 vector and the construction was checked by sequencing. The recombinant plasmid was transferred into *E*. *coli* BL21 DE3 strain. The recombinant *E*. *coli* strain was grown in LB medium and was induced at OD_600_ about 0.8 with IPTG (0.2 mg/mL) and then the culture was incubated at 20°C overnight with vigorous shacking. The cell pellet was sonicated in PBS with a cocktail of protease inhibitors and BoNT/E light chain was purified on cobalt column (Talon, Clontech) by elution with imidazole in PBS. The homogeneity of BoNT/E light chain preparation was checked on SDS-PAGE and assayed with the BioRad Bradford assay.

### Activation of BoNT/E3

BoNT/E3 was purchased as a single chain, and therefore proteolytic activation was performed prior to use to generate the active form. This also minimized the risk of denaturation of the activated BoNT/E3. Pure BoNT/E3 holotoxin (Metabiologics Inc.), 3 μg at a concentration of 1 μg/μL in PBS, was incubated for 30 min at 37°C with 0.15 μg trypsin (T1426, Sigma Aldrich). The cleavage reaction was stopped by adding 0.3 μg (100 μg/mL) trypsin inhibitor (T9003, Sigma Aldrich) and by incubating the reaction mixture for 30 min at 37°C. The extent of activation was assessed by capillary electrophoresis under reducing conditions where migration of a band corresponding to the uncleaved toxin (150 kDa) and the appearance of a 50 kDa and a 100 kDa band (data not shown) were monitored (Experion, Bio-Rad, Hercules, USA).

For *in vitro* endopeptidase inhibition studies, and *ex vivo* and *in vivo* neutralization studies concentrated pure hemagglutinin free BoNT/E3 at 1 mg/mL (3 x 10^5^ LD_50_/mg) was purchased from Metabiologics Inc. (Lot # E062805-01, strain Alaska). This toxin was trypsinised by using the method described by Jones et al., 2008 and activity determined as 6 x 10^7^ LD_50_/mg. After dilution to 12,560 LD50/mL in 50 mM gelatin (0.2% w/v) phosphate buffer, pH 6.5 toxin was stored frozen at -80°C and remained stable for several years.

### Animal immunization

**Fig 1:**
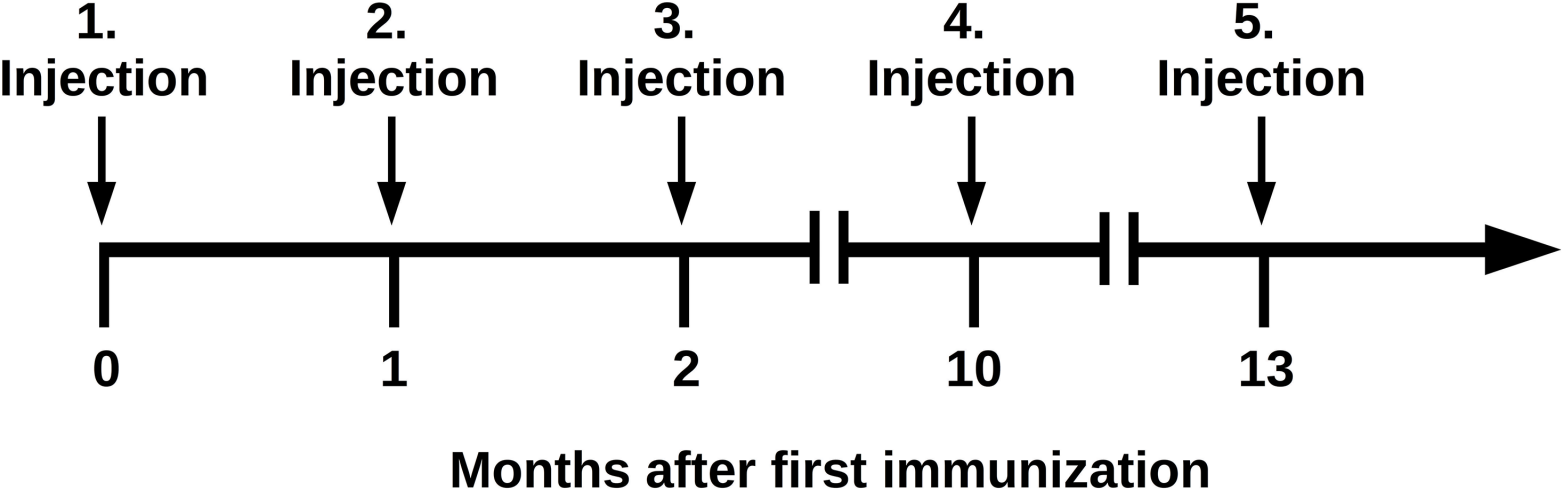
Schema of macaque immunization. Immunization was performed with five subcutaneous injections of recombinant BoNT/E3 light chain and sera were sampled to estimate the immunization titer.

A male cynomolgus macaque (*Macaca fascicularis*) was immunized with five subcutaneous injections of 80 μg recombinant botulinum neurotoxin light chain (BoNT/E3-L, amino acids M1 to G420). The first injection of BoNT/E was mixed with complete Freund’s adjuvant (Sigma, Isle d’ Abeau, France) and for the remaining injections, incomplete Freund’s adjuvant was used. The three first injections were administered at one-month intervals, the fourth injection was administered eight months after the third and the fifth was administrated three months after the fourth (Fig 1). The macaque immune response was evaluated by ELISA performed in 96-well microtiter plates (MaxiSorp, Nunc) using activated BoNT/E3 (2 μg/mL) in PBS. Pre-immune serum was used as a negative control. For detection, Fc-specific polyclonal anti-macaque immunoglobulin G (IgG) (Nordimmune, Tilburg, The Netherlands) was used and the titer was measured as the reciprocal of the highest dilution of immune serum giving a signal three times stronger than that of the negative control, at the same dilution.

### Construction of the anti-BoNT/E3-L scFv antibody library

After the fifth immunization, RNA was isolated with Tri Reagent^®^ (TR118, Molecular Research Center Inc.) from the bone marrow of the immunized macaque and used for reverse transcription. Seven oligonucleotides were used for amplification of the DNA encoding the κ light chain and nine oligonucleotides were used to amplify the DNA encoding Fd fragments of the γ chain [44]. To obtain two sub-libraries encoding the Fd fragment and κ light chain, the corresponding PCR products were pooled and sub-cloned into the pGEM^®^-T vector (A3600, Promega). The final library was constructed starting from these two sub-libraries. The DNA encoding the Fd fragment and the κ light chain was re-amplified with two individual oligonucleotide sets introducing the required restrictions sites for library-cloning using the pHAL35 vector. This vector is based on pHAL32 [38] and was designed for this library. Here, the tag order was changed from His/Myc to Myc/His, resulting in better expression of scFv in *E. coli [45]*. First the pHAL35 vector and the VL-amplified repertoire were digested with *Mlu*I and *Not*I (New England Biolabs). Afterwards, the enzyme reaction was terminated and the vector was dephosphorylated with calf intestinal phosphatase (MBI Fermentas). The vector and the VL-repertoire were purified using NucleoSpinII Gel and PCR Clean-up Kit (Macherey-Nagel) and 270 ng of the VL-repertoire was cloned into 1 μg of dephosphorylated vector separately in four ligation reactions using T4 DNA-Ligase (Promega). The DNA of the ligation product was precipitated with ethanol and sodium acetate. The pellet was washed twice with 70% ethanol and resuspended in 30 μL H_2_O before it was used for electroporation (1.7 kV) with 25 μL of XL1-Blue MRF’ (Stratagene). The transformed bacteria were cultured on 2xYT agar plates (25 cm petri dishes) supplemented with 100 μg/mL ampicillin, 20 μg/mL tetracycline and 100 mM glucose. The colonies were harvested by resuspension in 40 mL of 2xYT medium with a Drigalsky spatula and plasmids were isolated with the Nucleobond Plasmid Midi Kit (Macherey-Nagel). Following this, the VL-library and the VH-repertoire were digested with *Sfi*I and *Hind*III (New England Biolabs), ligated and electroporated as described for VL, but this time 250 ng of the digested and purified VH-repertoire was inserted into 1 μg of the VL-library. The harvested bacteria of the final scFv antibody gene library were pooled, aliquoted and stored at -80°C. The library was packaged with M13K07.

### Selection of BoNT/E3-L specific scFv by antibody phage display

For isolation of BoNT/E3-L specific scFv, a microtiter plate was coated overnight with activated holotoxin BoNT/E3 (Metabiologics Inc.) at 20 μg/mL in PBS at 4°C. The plate was then blocked with 3% BSA in TBS (50 mM TRIS; 137 mM NaCl; 2.7 mM KCl; pH adjusted to 8.0 with HCl) for 2 h at 37°C. After a washing step, the antibody phage display library was added and incubated for an additional 2 h at 37°C. During the first round of panning, the plate was washed 5 times with TBS supplemented with 0.1% Tween20^®^. Each wash consisted of pipetting up and down five times with an interval of five minutes between washes. The plate was finally rinsed with sterile TBS and phage were eluted with trypsin (10 mg/mL in TBS) for 30 min at 37°C. The eluted phage were used to infect *E*. *coli* (SURE strain, Stratagene) cultured in SB (Super Broth) supplemented with tetracycline (10 μg/mL) and carbenicillin (50 μg/mL). For the production of new phage particles, infected *E*. *coli* were co-infected with M13K07 and cultured overnight at 30°C in SB supplemented with tetracycline (10 μg/mL), carbenicillin (50 μg/mL) and kanamycin (70 μg/mL). Phage particles were precipitated in PEG/NaCl (4% (w/v) PEG–8000, 3% (w/v) NaCl) and used for the next round of panning. This was performed as described above, with the exception that the plate was washed 10 times during the second round, 20 times during the third and then 40 times in the fourth round, with TBS supplemented with 0.1% Tween20^®^ and a five-minute interval between washes. The infected *E*. *coli* of the fourth round of panning were grown on SB media in petri dishes and used for screening by ELISA using activated BoNT/E3 (2 μg/mL).

### DNA sequencing and sequence analysis

After screening, one hundred randomly chosen BoNT/E3 specific scFv-producing clones were sequenced by Beckman Coulter Genomics (Takeley, United Kingdom). All unique sequences were compared with the human germline genes using the IMGT/V-QUEST and IMGT/DomainGapAlign online tool from the International ImmunoGeneTics information system^®^ (IMGT) (http://www.imgt.org). This tool allows the identification of the human germline genes most similar to any given variable region and calculates the Germinality Index (GI), defined as the percentage identity between a given framework (FR) and the most similar human germline sequence.

### Production of soluble scFv or scFv-Fc and measurement of specific binding and affinity

For expression of soluble scFv, the DNA encoding the selected scFv was used to transform the non-suppressor *E*. *coli* strain HB2151 [46]. Transformed *E*. *coli* were then used to inoculate 5 mL of SB (Super Broth) medium supplemented with carbenicillin (50 μg/mL) and 1% glucose. Cultures were incubated overnight at 30°C with shaking (250 rpm). The following day, 500 mL of SB medium (50 μg/mL carbenicillin, 1% glucose) was inoculated with the overnight culture (OD_600_ ~ 0.1) and cultivated at 30°C until OD_600_ ~ 1.5. Then, 1 mM IPTG was added to induce the expression of scFv and the culture was incubated overnight at 22°C. After harvesting by centrifugation at 2,500 x g for 15 min at 4°C scFv were extracted with polymyxin B sulfate and purified using a HIS Trap column (GE Healthcare, Buckinghamshire, UK) by a Profinia automat (Bio-Rad, Hercules, California) according to the manufacturer's instructions. Purified scFv were quantified using capillary electrophoresis on an Experion device (Bio-Rad).

All scFv fragments were subcloned into pCSE2.5-mIgG2c-Fc-XP (murine Fc part consisted of CH2 and CH3) and produced as scFv-Fc in HEK293–6E cells [National Research Council (NRC), Biotechnological Research Institute (BRI)] cultured in chemically defined medium F17 (Invitrogen, Life Technologies) supplemented with 1 g/L pluronic F68 (Applichem), 4 mM L-glutamine (PAA) and 25 mg/L G418 (PAA), as previously described [47]. DNA was used for the transient transfection of 25 mL cultures of HEK293–6E cells in 125 mL Erlenmeyer shake flasks. After 48 h of culture with shaking at 110 rpm in a Minitron orbital shaker (Infors) at 37°C, under an atmosphere containing 5% CO_2_, one volume of culture medium, with a final concentration of 0.5% (w/v) tryptone N1 (TN1, Organotechnie S.A.S.) was added and cultivated for an additional 3 days. ScFv-Fc were purified with a UNOsphere SUPrA column (Biorad) using a Profinia apparatus (Biorad, Hercules), according to the manufacturer’s instructions.

For ELISA, a microtiter plate was coated overnight with activated holotoxin BoNT/E3 (Metabiologics Inc.) at a concentration of 20 μg/mL in PBS at 4°C. The wells were blocked by incubation with 2% (w/v) skim milk powder in PBS supplemented with 0.1% Tween20^®^ (2% MPBST) for 1.5 h at room temperature, and washed three times with PBS supplemented with 0.1% Tween20^®^ (PBST). Soluble scFv were diluted in 100 μL 2% MPBST and were incubated in the antigen-coated wells for 1.5 h at room temperature. The wells were then washed three times with PBST and bound scFv were detected with an anti-His tag antibody (QIAGEN, Courtaboeuf, France).

Antibody affinity was determined by surface plasmon resonance spectroscopy using a BIAcore 3000 instrument (GE Healthcare-Biacore, Uppsala, Sweden). ScFv were coated at a maximum of 800 RU on a CM5 chip (BR100012, GE Healthcare-Biacore) via amine coupling according to the manufacturer's instructions. A flow rate of 30 μL/min was maintained during each measurement and volumes of 100 μL of at least ten dilutions of activated BoNT/E3 (250 nM to 0.03 nM) dissolved in HBS-EP buffer (BR100188, GE Healthcare-Biacore) were tested. After each dilution, the chip was regenerated with 1.5 μM glycine buffer (BR100354, GE Healthcare-Biacore) injected for 30 s at 10 μL/min. Affinities were calculated using the BIAevaluation software (GE Healthcare-Biacore) according to the Langmuir 1:1 adsorption model and were verified by internal consistency test [48].

### *In vitro* endopeptidase immunoassay

The *in vitro* endopeptidase immunoassay for BoNT/A and BoNT/E have been reported previously [49] The assay was modified to assess the inhibitory properties of the scFv and scFv-Fc [38,50]. For BoNT/E endopeptidase assay synthetic 70 aa SNAP–25 (137–206) peptide substrate was purchased from Immune Systems Ltd (Paignton, UK) and BoNT/E cleavage site specific antibody (SNAP–25 173–180) was made in house in rabbits and used at 40 μg/mL as previously described [49]. Inhibition studies were performed to determine the concentrations at which scFv fragments could inhibit the endopeptidase activity conferred by a fixed concentration of BoNT/E toxin. This was selected as the concentration producing approximately 80–90% of maximum activity in dose-response study. This was 20LD_50_/mL (330 pg) for pure holotoxin BoNT/E. A range of concentrations of scFv were mixed with an equal volume of 40LD_50_/mL of pure holotoxin BoNT/E (75 μL + 75 μL) to give a final concentration of 20LD_50_/mL (330 pg) of BoNT/E toxin and a range of concentration of scFv (2.58 to 660 nM). The toxin/scFv mixture was briefly shaken on a plate shaker for 1 min at room temperature and the reaction mixture was incubated for 1 h at 37°C prior to transfer (50 μL/well) to polystyrene 96 well ELISA plates coated with SNAP–25 (137–206) peptide for estimation of toxin endopeptidase activity. All dilutions were made in reaction buffer (50 mM HEPES, 20 μM ZnCl_2_, pH 7.0, 0.5% (v/v) Tween20^®^, 5 mM DTT) and incubated for 18 h at room temperature. Washes were performed three times with PBS supplemented with 0.05% (v/v) Tween20^®^. Reaction of non-inhibited BoNT/E toxin on SNAP–25 (137–206) peptide was detected by anti-SNAP–25 (173–180) antibody at 40 μg/mL diluted in 2.5% (w/v) skimmed milk powder in phosphate buffered saline (PBS). After 1 h incubation at room temperature, the plate was washed with PBS supplemented with 0.05% (v/v) Tween20^®^ and 100 μL per well of goat anti-rabbit (Sigma A0545) detection antibody (1:2000 diluted in 2.5% MPBST) were added and the plate incubated again for 1.5 h. at room temperature. After washing, 100 μL per well substrate solution (50 mM citric acid pH 4.0, 0.05% (w/v) ABTS (2,2’-azino-bis(3-ethylben-zothiazoline-6-sulfonic acid) diammonium salt, and 0.05% (v/v) of a 30% (w/v) hydrogen peroxide solution) were added and the staining reaction was evaluated by determining absorbance at 405 nm with a plate reader (Multiskan MS, Labsystems). To allow meaningful comparison, all scFv antibody fragments were diluted to the same initial concentration of 40 μg/mL and doubling serial dilutions were made from this concentration prior to adding BoNT/E.

### *Ex vivo* mouse phrenic nerve-hemidiaphragm assay

Left phrenic nerve-hemidiaphragm preparations were excised from male inbred mice (BALB/c) and installed in a 6 mL organ bath maintained at 37°C containing Krebs-gelatin buffer gassed with 95% O_2_-5% CO_2_ bubbled through the buffer. Indirect stimulation via the phrenic nerve was achieved with a supramaximal voltage (~3 V, 1 Hz, 0.2 ms) and the resulting muscle contractions were measured with an isometric force transducer (FMI GmbH) linked to a ML110 bridge amplifier and a Powerlab/4SP 4 channel recorder (ADInstruments). The hemidiaphragm resting tension was increased in a stepwise manner during the equilibration period, until reproducible twitches were observed. The toxin was diluted in oxygenated Krebs buffer and incubated at 37°C for 30 min before being applied to the tissue. Once the muscle twitch response to nerve stimulation had stabilized and remained of a constant magnitude for at least 30 min without further adjustment, the Krebs buffer was replaced with 6 mL of toxin in Krebs solution and stimulation was resumed. Toxin-induced paralysis was defined as a 50% decrease in the muscle twitch response to neurogenic stimulation, based on the magnitude of the contractions just before the addition of the toxin. The time to 50% paralysis after the addition of BoNT/E3 was determined by fitting to the linear part of the paralysis curve. The myotoxic effects of the toxin were also assessed by applying a short burst of direct (muscle) stimulation (~30 V, 1 Hz, 1 ms,) before adding the toxin and at the end of the experiment. Toxin neutralization by the antibody preparations was assessed by mixing 330 pg/mL (20LD_50_/mL) pure BoNT/E3 holotoxin with different concentrations of antibody preparation and incubating for 30 min at 37°C before applying the mixture to the tissue. The toxin dose considered optimal for antibody inhibition studies is typically that inducing about 80% maximum paralytic activity on the dose-response curve (ensuring optimal precision), unless further antibody characterization requires weaker paralytic activity. The organ bath was drained, the toxin/antibody mixture was added and twitch responses were recorded for up to 300 min (5 h) or until the twitch tension was no longer detectable. The time to 50% paralysis after the addition of the mixture of BoNT/E3 and antibody was determined by fitting to the linear part of the paralysis curve [51,52]. Linear and nonlinear regression analyses were performed with Prism 5.0 software (Graphpad). The neutralization of toxin activity was proportional to the ability of the antibody to delay BoNT-induced paralysis. Greater antibody neutralizing potency was associated with the requirement of a longer period of time for the hemidiaphragm to reach 50% paralysis for the same dose of toxin.

### *In vivo* neutralization studies in mouse flaccid paralysis assay

Botulinum toxin induces asymmetric flaccid muscular paralysis at the site of injection and this has formed the development of an assay for toxin and serotype specific neutralization. The method was further validated against the mouse LD_50_ test for neutralization of BoNT/A by specific IgG and its smaller fragments.

Neutralization activity of scFv-Fc ELC18 (selected as described above) was assessed *in vivo* using a range of doses of the antibody (from 1.0 μg per dose). BoNT/E3 toxin (Metabiologics pure toxin was used at 1LD_50_ (24 pg) per dose. All dilutions were prepared in gelatin (0.2% w/v) phosphate (50 mM di-sodium hydrogen orthophosphate) buffer, (GPBS, pH 6.5), and toxin: antibody mixtures were left for 30 min at room temperature before injecting 0.1 mL subcutaneously into female MF1 strain of mice, weighing between 16–20 g (n = 4 per dose). All injections were performed within 30 min and all the mice were injected in the left inguinocrural region. Scores were recorded at 24 h post injection, with intensity of paralysis ranging from 0 (no sign of paralysis), to scores between 1 and 4, defined by an increasing extent of local flaccid paralysis. A positive control group of mice were injected with BoNT/E3 toxin alone, and a negative control group of mice were injected with the maximum concentration of antibody used in the assay in the absence of toxin.

## Results

### Animal immunization and construction of the antibody phage display library-cloning

As shown in Fig 1 a male macaque (*Macaca fascicularis*) was immunized with five injections of recombinant BoNT/E3 light chain (BoNT/E3-L). Fourteen days after the second injection, the immune response was evaluated by ELISA using BoNT/E3 holotoxin and a titer equal to 1:300,000 was observed. Three months after the fourth injection, bone marrow was sampled but no RNA encoding the Fd fragments and the κ light chain were amplified using specific oligonucleotides. Three months after the fourth injection, a fifth injection with BoNT/E3-L was administered. The bone marrow was iteratively sampled over 17 days, and the bone marrow sampled at the 10^th^ day after the last immunization provided the optimal amplification of the DNA encoding the Fd fragments and the κ light chain. The PCR-products were pooled and sub-cloned into pGEM^®^-T vector to obtain two sub-libraries of 4.1 x 10^4^ and 2.3 x 10^4^ clones encoding the Fd fragments and κ light chains, respectively. For final construction of the immune library, the phage display vector pHAL35 was used. This vector is a modified form of pHAL32 with changed His/Myc-tag orientation, which results in a better production-rate of soluble scFv [45]. The scFv library was constructed by two consecutive cloning steps. First, the κ light chain was cloned, using *Mlu*I and *Not*I, and then the Fd fragments, using *Sfi*I and *Hind*III. The final scFv antibody gene library consisted of 9.6 x 10^8^ independent clones with a full-size insert rate of 88%. Finally, the immune library was packaged with M13K07, resulting in phage titers of 3.4 x 10^13^ cfu/mL and this was used for antibody selection.

### Isolation of BoNT/E3-L specific scFv

A multi-step panning of four rounds was performed against BoNT/E3 holotoxin with 5, 10, 20 and 40 washes, respectively. After the third round of panning 1.6 x 10^6^ scFv-phage were isolated. After each round of panning, the reactivity of the eluted phage against BoNT/E3 was assessed by phage-ELISA and the signal increased 1.5-fold between round 1 and 4. In addition, no reactivity against trypsin and trypsin inhibitor, nor against BSA, KLH and LF was observed (data not shown). After the fourth round of panning, one hundred scFv-clones were randomly chosen, the scFv encoding DNA was extracted and sequenced. Only five sequences appeared to be recombined and the corresponding clones were discarded. Of the 95 remaining scFv, 78 sequences were identified in a single copy and seven were identified in two or more copies, resulting in a total of 85 different scFv sequences. The diversity was represented in the form of a phylogenetic tree and at least one sequence from each cluster was selected resulting in the selection of 38 scFv. After transformation of the DNA in HB2151 all 38 scFv were successfully expressed and purified.

### Analysis of the represented germline genes

After sequencing, analysis using IMGT/V-QUEST tool was performed, to identify the human germline V, (D), J genes most similar to the 38 selected scFv that were selected for further *in vitro* analysis (Table 1). Almost all heavy chain V genes were dominated by the IGHV4 gene family, with the exception of 2 occurrences of the IGHV1 and IGHV3 gene family. These genes were combined with representatives of all IGHD genes and IGHJ genes. The diversity of the light chain was limited due to the presences of mostly IGKV1 genes with only one occurrence of IGKV7. These genes were combined with nearly all IGKJ genes. The Germinality Index (GI) for each VH and VL was calculated using IMGT/DomainGapAlign and provided an indication of the identity between framework regions of the selected scFv and those encoded by the most similar human germline genes, as a percentage. The average GI-values for all 38 scFv were 86.9% (VH) and 89.4% (VL).

**Table 1:** Human germline genes most similar to the genes encoding the BoNT/E3 specific scFv.

scFv	VH			VL		GI [%]	
	V	D	J	V	J	VH	VL
ELC7	IGHV4-39*07	IGHD2-15*01	IGHJ5*01	IGKV1-17*01	IGKJ4*01	89.0	92.1
ELC8	IGHV4-59*02	IGHD1-26*01	IGHJ4*02	IGKV1-16*01	IGKJ3*01	85.7	84.3
ELC9	IGHV4-39*07	IGHD2-15*01	IGHJ5*01	IGKV1-6*01	IGKJ4*01	87.9	91.0
ELC11	IGHV4-39*07	IGHD3-3*01	IGHJ5*01	IGKV1-17*01	IGKJ2*01	85.7	92.1
ELC12	IGHV4-39*07	IGHD3-22*01	IGHJ5*01	IGKV1-17*01	IGKJ4*01	89.0	94.4
ELC14	IGHV4-28*01	IGHD3-22*01	IGHJ3*01	IGKV1-17*01	IGKJ2*03	86.8	88.8
ELC18	IGHV4-28*01	IGHD2-21*01	IGHJ3*01	IGKV1-9*01	IGKJ2*03	85.7	89.9
ELC20	IGHV3-NL1*01	IGHD1-20*01	IGHJ4*03	IGKV1D-13*01	IGKJ1*01	81.3	87.6
ELC21	IGHV4-39*07	IGHD4-11*01	IGHJ4*02	IGKV1D-16*01	IGKJ3*01	89.0	85.4
ELC22	IGHV4-38-2*01	IGHD2-8*02	IGHJ5*01	IGKV1-17*01	IGKJ4*01	85.7	92.1
ELC24	IGHV4-59*02	IGHD2-2*01	IGHJ4*02	IGKV1-12*01	IGKJ2*03	89.0	83.1
ELC25	IGHV4-39*07	IGHD2-8*02	IGHJ5*01	IGKV1-6*01	IGKJ4*01	85.7	93.3
ELC31	IGHV3-66*02	IGHD2-21*02	IGHJ5*02	IGKV1-5*03	IGKJ3*01	89.0	85.4
ELC32	IGHV4-38-2*01	IGHD2-8*02	IGHJ5*01	IGKV1-17*01	IGKJ4*01	84.6	94.4
ELC37	IGHV4-28*01	IGHD2-21*01	IGHJ3*01	IGKV1-17*01	IGKJ3*01	83.5	89.9
ELC40	IGHV4-59*02	IGHD1-26*01	IGHJ4*02	IGKV1-5*03	IGKJ2*03	85.7	86.5
ELC41	IGHV1-2*02	IGHD5-12*01	IGHJ5*02	IGKV7-3*01	IGKJ3*01	82.4	75.3
ELC45	IGHV4-39*07	IGHD1-26*01	IGHJ5*02	IGKV1-9*01	IGKJ2*03	85.7	91.0
ELC51	IGHV4-39*07	IGHD2-15*01	IGHJ5*01	IGKV1-17*01	IGKJ3*01	89.0	83.2
ELC55	IGHV4-39*07	IGHD1-26*01	IGHJ5*02	IGKV1-9*01	IGKJ2*03	89.0	91.0
ELC58	IGHV4-30-4*07	IGHD6-13*01	IGHJ4*02	IGKV1-12*01	IGKJ2*01	82.4	89.9
ELC62	IGHV4-30-4*07	IGHD6-25*01	IGHJ3*02	IGKV1-12*01	IGKJ4*01	85.7	87.6
ELC63	IGHV4-28*01	IGHD3-22*01	IGHJ5*02	IGKV1-16*01	IGKJ2*03	91.2	91.0
ELC65	IGHV4-28*01	IGHD2-21*02	IGHJ4*02	IGKV1-6*01	IGKJ2*03	91.2	84.3
ELC69	IGHV4-30-4*07	IGHD1-20*01	IGHJ1*01	IGKV1-16*01	IGKJ4*01	94.5	88.8
ELC72	IGHV1-69-2*01	IGHD3-10*01	IGHJ2*01	IGKV1-16*01	IGKJ4*01	84.6	92.1
ELC73	IGHV4-39*07	IGHD2-15*01	IGHJ5*01	IGKV1-17*01	IGKJ2*01	87.9	84.3
ELC76	IGHV4-39*07	IGHD2-15*01	IGHJ5*01	IGKV1-6*01	IGKJ4*01	87.9	94.4
ELC77	IGHV4-30-2*01	IGHD5-12*01	IGHJ1*01	IGKV1-16*01	IGKJ4*01	90.1	91.0
ELC78	IGHV4-4*02	IGHD4-17*01	IGHJ1*01	IGKV1-16*01	IGKJ3*01	89.0	93.3
ELC81	IGHV4-59*02	IGHD3-10*02	IGHJ4*02	IGKV1-12*01	IGKJ4*01	89.0	85.4
ELC88	IGHV4-28*01	IGHD3-9*01	IGHJ4*02	IGKV1-17*01	IGKJ2*01	78.0	91.0
ELC92	IGHV4-30-2*01	IGHD5-12*01	IGHJ1*01	IGKV1-9*01	IGKJ1*01	92.3	92.1
ELC95	IGHV4-30-2*01	IGHD5-24*01	IGHJ4*02	IGKV1-12*01	IGKJ3*01	84.6	92.1
ELC96	IGHV4-39*07	IGHD4-11*01	IGHJ4*02	IGKV1-12*01	IGKJ3*01	89.0	93.3
ELC97	IGHV4-4*02	IGHD1-1*01	IGHJ5*01	IGKV1-39*01	IGKJ4*01	85.7	87.6
ELC103	IGHV4-28*01	IGHD3-22*01	IGHJ3*01	IGKV1-9*01	IGKJ2*03	86.8	94.4
ELC109	IGHV4-30-4*07	IGHD6-13*01	IGHJ4*02	IGKV1-16*01	IGKJ2*03	81.3	93.3

The human germline genes most similar to the genes encoding the 38 selected scFv were identified by IMGT/V-QUEST analyses. The Germinality Index (GI) of each scFv (VH and VL) was calculated using IMGT/DomainGapAlign.

### *In vitro* endopeptidase immunoassay

**Fig 2:**
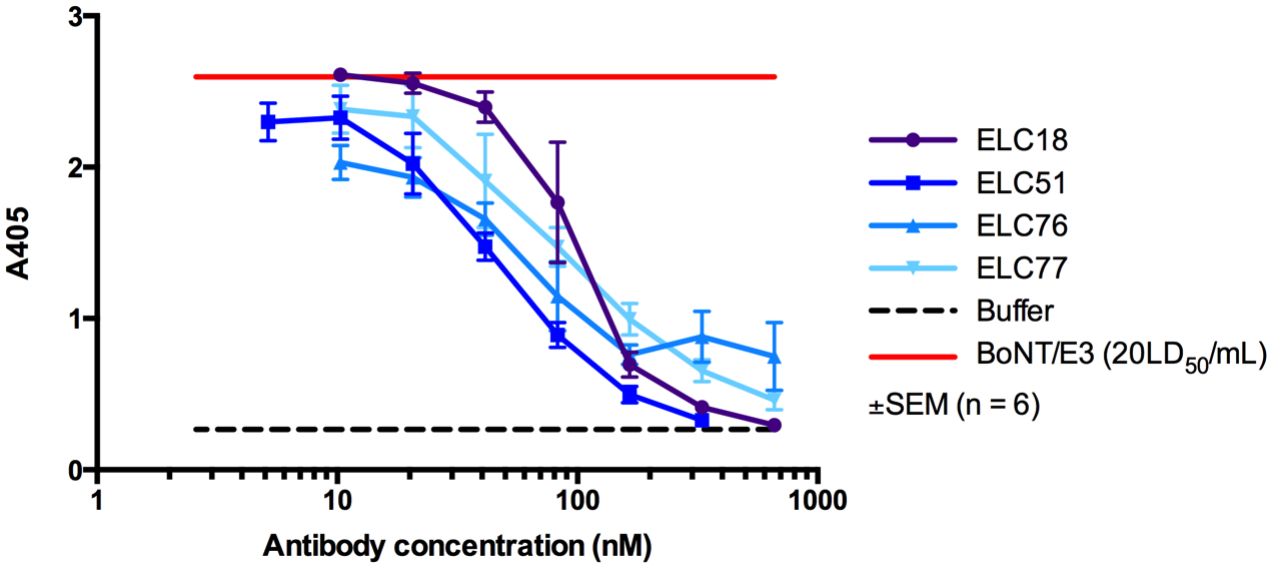
*In vitro* endopeptidase assay. Concentration-dependent inhibition of the endopeptidase activity of BoNT/E3 holotoxin (330 pg/mL; 20LD_50_/mL) *in vitro* by several scFv targeting the light chain of BoNT/E3. As control, SNAP–25 cleavage by BoNT/E3 without inhibiting scFv and buffer without toxin. Each data point is a mean of triplicate determinants from two separate experiments (n = 6) ± SEM.

**Fig 3:**
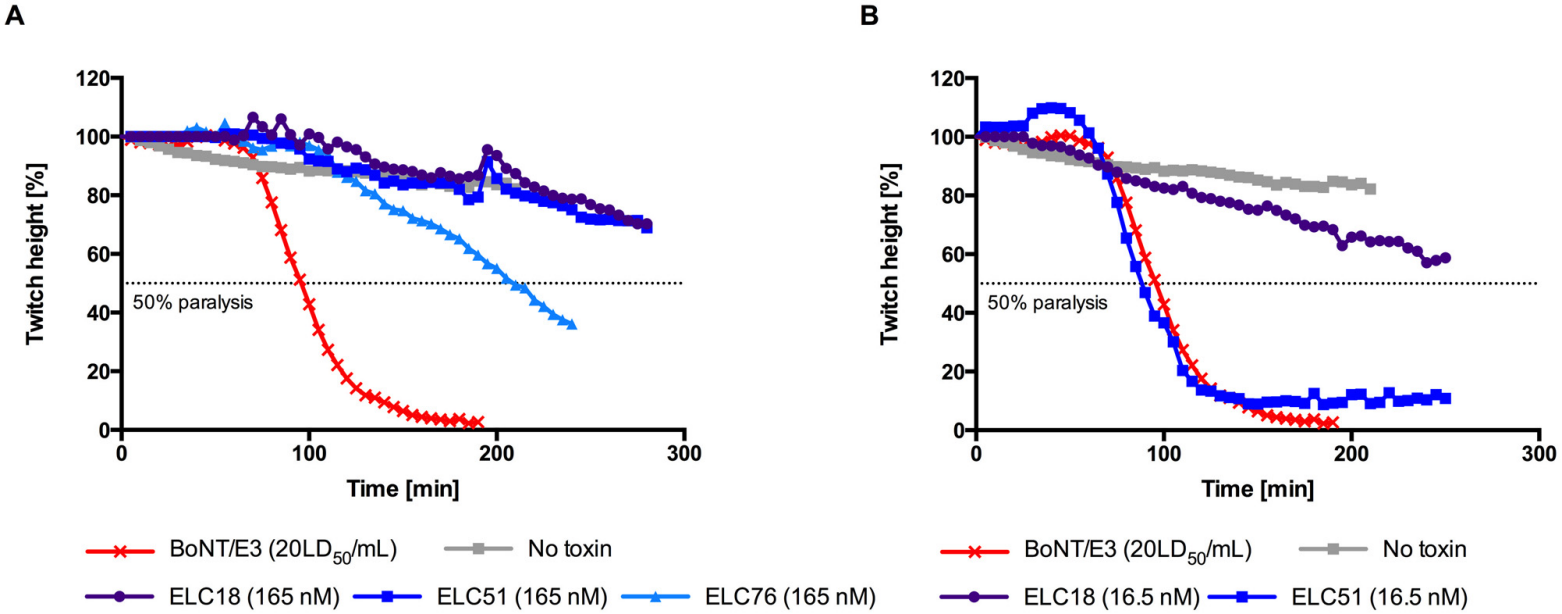
*Ex vivo* mouse phrenic nerve-hemidiaphragm assay. A) Neutralization of BoNT/E3 holotoxin (330 pg/mL; 20LD_50_/mL) by 165 nM scFv. B) Neutralization of BoNT/E3 holotoxin (330 pg/mL; 20LD_50_/mL) by 16.5 nM scFv of ELC18 and ELC51. Successful neutralization resulted in a longer 50% paralysis time of the hemidiaphragm at the same dose of toxin. As a control, a hemidiaphragm preparation exposed only to Krebs, without toxin, was used. Each data set is from a single hemidiaphragm preparation.

**Fig 4:**
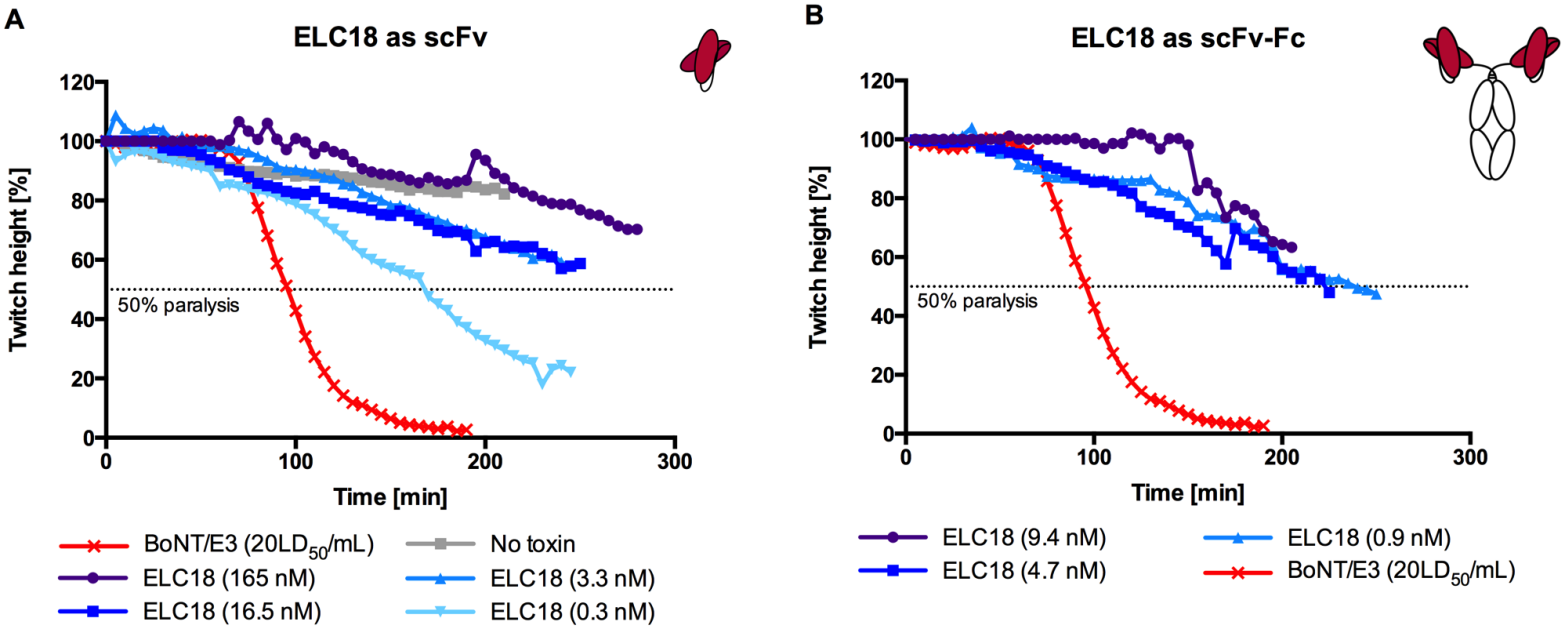
*Ex vivo* mouse phrenic nerve-hemidiaphragm assay with ELC18. A) Neutralization of BoNT/E3 holotoxin (330 pg/mL; 20LD_50_/mL) by different concentrations of ELC18. Neutralization properties of ELC18 as scFv at 165 nM, 16.5 nM, 3.3 nM, and 0.3 nM were evaluated. B) Neutralization properties of ELC18 as scFv-Fc at 9.4 nM, 4.7 nM, and 0.9 nM were evaluated. Each neutralization set is from a single hemidiaphragm preparation per concentration.

**Fig 5:**
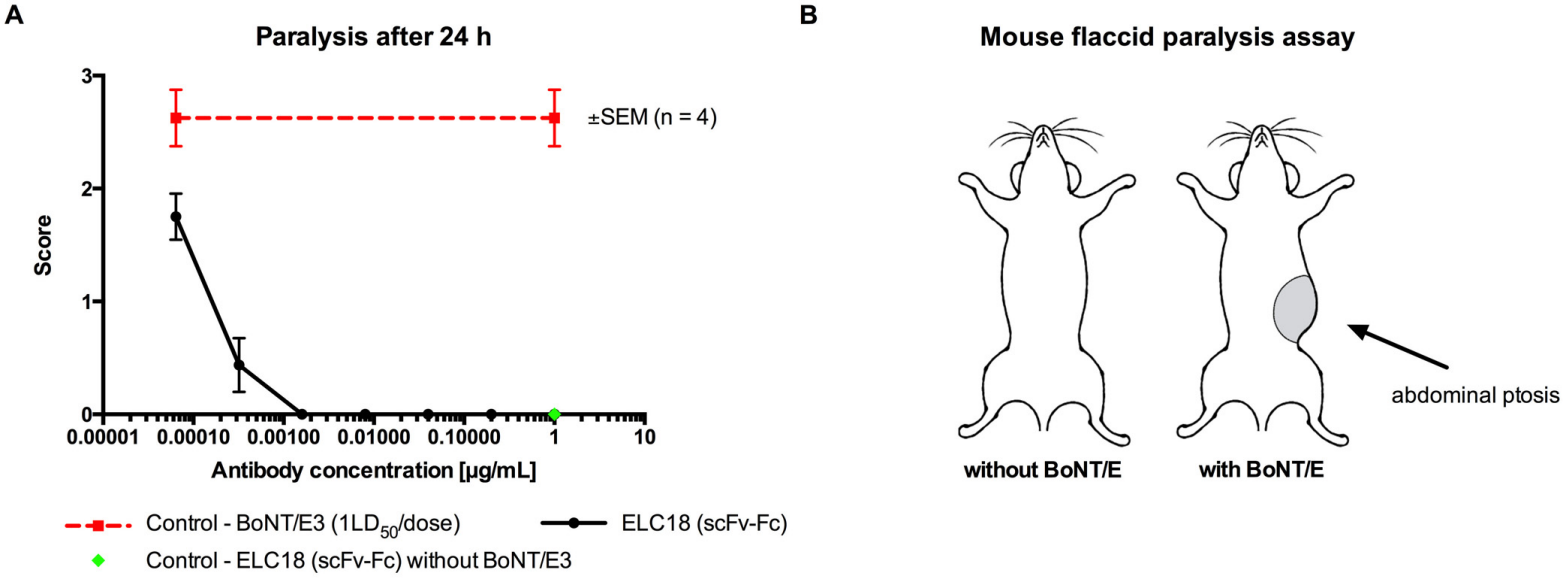
*In vivo* mouse paralysis assay with ELC18 as scFv-Fc. A) The neutralization activity of ELC18 was determined in the mouse paralysis assay *in vivo*. BoNT/E3 holotoxin (1LD_50_ per dose) was pre-mixed with each antibody at 0.064 ng, 0.32 ng, 1.6 ng, 8 ng, 40 ng, 0.2 μg and 1 μg per dose. Animals were scored at 24 h post injection. Results are expressed as mean score for 4 mice ± SEM. Positive control group of mice were injected with BoNT/E3 toxin alone and negative control group of mice received the maximum concentration of antibody in the absence of toxin. B) Schema of the *in vivo* mouse paralysis assay. Formation of abdominal ptosis after injection of botulinum neurotoxin.

The 38 selected scFv were assessed for their inhibition capacities in the SNAP–25 endopeptidase immunoassay. Four scFv effectively inhibited BoNT/E3 holotoxin with antibody concentrations producing 50% BoNT/E3 inhibition (IC_50_ values) of 33.8 nM (ELC76), 49.1 nM (ELC77), 58.6 nM (ELC51) and 112.8 nM (ELC18), respectively (Fig 2, Table 2) (The raw data for Figs 2–5 are given in S1 File). The most potent inhibitor, ELC76, inhibited the endopeptidase activity of BoNT/E3 at a molar ratio of scFv to toxin of 14,765:1 (IC_50_ of 33.8 nM) (Table 2). The scFv inhibiting toxin endopeptidase activity were selected for further analyses using the *ex vivo* mouse phrenic nerve-hemidiaphragm assay and affinity measurement.

**Table 2:** Inhibition of BoNT/E3 endopeptidase activity.

scFv	Antibody concentration for 50% endopeptidase inhibition (IC_50_) [nM]	Molar ratio scFv:toxin
ELC18	112.8	49,214
ELC51	58.6	25,571
ELC76	33.8	14,765
ELC77	49.1	21,428

### Affinity measurement

The affinity of the four scFv that inhibited the endopeptidase activity of BoNT/E3 was determined by surface plasmon resonance spectroscopy. All affinities were in the sub-nanomolar range from 0.01 nM (ELC77) to 9.16 nM (ELC51) (Table 3). The four selected scFv did not react against trypsin or trypsin-inhibitor (data not shown). These antigens were used as controls as they were part of the antibody selection process for toxin activation. No correlation was observed between affinity and IC_50_.

**Table 3:** Affinities of the four inhibiting scFv.

scFv	K_off_ [s^−1^]	K_on_ [M^−1^s^−1^]	K_D_ [nM]
ELC18	1.94 x 10^−5^	3.37 x 10^4^	0.58
ELC51	4.37 x 10^−5^	4.77 x 10^3^	9.16
ELC76	3.56 x 10^−6^	3.45 x 10^3^	1.03
ELC77	1.10 x 10^−5^	1.16 x 10^5^	0.01

### *Ex vivo* mouse phrenic nerve-hemidiaphragm assay

To confirm that the antibodies that inhibited the endopeptidase activity of BoNT/E3 *in vitro* also have toxin neutralization properties, their neutralization capacity against 20LD_50_/mL BoNT/E3 was tested at a concentration of 165 nM in the *ex vivo* mouse phrenic nerve-hemidiaphragm assay (Fig 3A, Table 4). Only ELC18, ELC51 and ELC76 were tested *ex vivo*. Sequence analysis of ELC77 showed a glycosylation site and it was discarded from further development. As controls phrenic nerve-hemidiaphragm preparations incubated in Krebs with or without BoNT/E3 were used for negative and positive comparison of the antibody induced neutralization (Table 4). At a concentration of 165 nM ELC18 and ELC51 showed a similar neutralization efficacy with 50% paralysis time of 445 min for ELC18. The 50% paralysis time of BoNT/E3 without antibodies (20LD_50_/mL) was 95 min. For further analysis the assay was repeated with 16.5 nM scFv concentration (Fig 3B). At this concentration, only ELC18 was neutralizing. Subsequently, only ELC18 was further analyzed at lower concentrations (Fig 4A, Table 5). Significant neutralization (at least 25% inhibition of activity, 164 min paralysis time) was confirmed with as low as 0.3 nM scFv ELC18, and almost 50% inhibition occurred at ~3.3 nM. This represents more favorable neutralization than inhibition *in vitro*, with a molar ratio of ~1,500:1, an increase of 33-fold. For further analysis, ELC18 was converted into the scFv-Fc antibody format and tested at different dilutions (9.4 nM, 4.7 nM and 0.9 nM) in the *ex vivo* paralysis assay (Fig 4B). At the lowest concentration of 0.9 nM, a 50% paralysis time of 237 min was achieved, representing a molar ratio (scFv-Fc:toxin) of ~393:1.

**Table 4:** *Ex vivo* phrenic nerve-hemidiaphragm assay. Neutralization of BoNT/E3 toxicity (20LD_50_/mL) by the BoNT/E3 specific scFv ELC18, ELC51 and ELC76.

scFv	50% paralysis time [min]
ELC18 (165 nM)	445
ELC51 (165 nM)	432
ELC76 (165 nM)	210
BoNT/E3 (20LD_50_/mL)	95
No toxin	(536*)

* Extrapolation

**Table 5:** *Ex vivo* phrenic nerve-hemidiaphragm assay. Neutralization of BoNT/E3 toxicity (20LD_50_/mL) by the BoNT/E3 specific ELC18 as scFv at different concentrations.

ELC18 (scFv)	50% paralysis time [min]
165 nM	445
16.5 nM	290
3.3 nM	291
0.3 nM	164

### *In vivo* neutralization of scFv-Fc against BoNT/E

The scFv-Fc form of ELC18 targeting BoNT/E-L was tested *in vivo* in the non-lethal mouse flaccid paralysis protection model against pure BoNT/E3 (1LD_50_ per dose). Results after 24 h are reported. A range of 6 doses of antibody, starting from 1.0 μg per dose, was used. Complete protection was achieved with 1.6 ng of ELC18, representing a molar ratio (antibody:toxin) of ~87:1. Partial protection was observed with two further 5-fold dilutions (Fig 5).

## Discussion

Botulinum neurotoxins (BoNTs) are the most toxic substances known and cause botulism, a life-threatening disease characterized by flaccid muscle paralysis. Recombinant antibodies are of particular interest to neutralize biological warfare agents as *Bacillus anthracis*, ricin or BoNTs. They can be used for therapy and prophylaxis for personals at high exposure risk [11,53]. To our knowledge, no recombinant human-like antibody neutralizing BoNT/E by targeting the light chain (BoNT/E-L) has been reported to date. Only neutralizing antibodies targeting the heavy chain of BoNT/E were described, such as 4E17 isolated from a human immune library and targeting a conserved epitope of the BoNT translocation domain [40], or BMR2, a single domain VHH from dromedary recognizing the binding domain of BoNT/E [54]. In this study we focused on BoNT/E3 and described the isolation of several different scFv which neutralized BoNT/E3 by targeting its light chain. Non-human primates are phylogenetically close to humans and their antibody sequences are similar to those in humans [55]. This is important to ensure that any antibody isolated are well tolerated in therapeutic applications. Macaques have been used for immunization with antigens of biodefense interest, without untoward effects [56,57]. In this study, after immunization of a macaque an antibody phage display library was constructed as previously described [37,35,31,33,38]. For immunization, only the non-toxic recombinant light chain of BoNT/E3 was used to induce a hyper-immune response. However, in validation of the immune response, antibody phage screening, affinity measurement, *in vitro*, *ex vivo* and *in vivo* assays were all based on the holotoxin to ensure that only scFv that recognize the complete toxin were selected. The constructed scFv library comprised of 9.6 x 10^8^ clones with a full-sized scFv insert rate of 88%, which is similar to previously published similar studies isolating neutralizing scFv with nanomolar affinities [35,38]. Finally, 38 scFv considered as representatives of the sequence diversity were selected, then expressed in *E*. *coli* and tested in the SNAP–25 endopeptidase assay [49]. Four scFv were identified, that inhibited the endopeptidase activity of BoNT/E3 *in vitro*. The best way to compare the inhibitory effectiveness of these antibodies is to use the molar ratio of antibody to toxin at which 50% inhibition was observed. ELC76 was the most effective scFv with an IC_50_value of 33.8 nM and a molar ratio of 14,765:1 (scFv:toxin). Affinity of the four endopeptidase-inhibiting scFv was measured by surface plasmon resonance. ELC77 had the highest affinity, 0.01 nM, whereas the affinity of ELC18 was 0.58 nM. For a more realistic evaluation of the antibodies an assay that addresses their neutralization capacity was needed. Here, the *ex vivo* mouse phrenic nerve-hemidiaphragm assay was chosen. This *ex vivo* assay is closely mimicking the *in vivo* respiratory paralysis caused by BoNTs [51] and *ex vivo* neutralization assessments highly correlated with those of the *in vivo* assay for assessment of the neutralization activity of polyclonal antibodies against BoNT serotypes A, B and E [52]. The most effective neutralization was achieved with ELC18, with neutralization at a molar ratio of ~1,500:1. This is about 33-times more favorable than *in vitro* inhibition of light chain activity. There was, no relationship between affinity, neutralization capacity and IC50 inhibition, indicating that the selected antibodies bind to different epitopes. Compared to scFv, IgG has a higher steric hindrance, so that converting the scFv to full-length IgG should improve the IC_50_ value and the molar ratio. As an example, the molar ratio for 2H8, an scFv antibody neutralizing BoNT/A1 (20LD_50_/mL) in a similar endopeptidase assay to that used here by targeting the light chain, was 64,000:1 (scFv:toxin). After converting 2H8 into a scFv-Fc format, a bivalent antibody similar to IgG, 50% inhibition was observed at a molar ratio of 6,500:1 (scFv-Fc:toxin) [38]. After conversion into scFv-Fc, ELC18 was effective at neutralizing BoNT/E3 in the *ex vivo* assay at 0.9 nM, representing a molar ratio of ~393:1, an improvement on the scFv. More importantly, when tested *in vivo* ELC18 scFv-Fc was protective at 1.6 ng/dose against 1LD_50_ BoNT/E3, representing a molar ratio of only ~87:1.

For further therapeutic development, ELC18 should be validated as IgG in lethal assays. IgG 4E17.1, an affinity matured variant of 4E17, protected mice challenged with 200LD_50_/mL of BoNT/E at a single dose of 25 μg IgG [40]. Unlike scFv produced in bacterial cells, mammalian cells that are known to glycosylate protein, so that any glycosylation of the variable region of IgG produced in such cells could hinder antigen binding. ELC77 was predicted to be glycosylated from its sequence and was therefore discarded from further development, to avoid any decrease in affinity or in neutralization capacity after expression as full length IgG in an eukaryotic system. For human treatment, the potential immunogenicity of a recombinant antibody is of concern. It is considered that more human-like antibodies are well tolerated, because they should be considered as a part of the immunological-self [55,58,59]. Nevertheless, to limit the risk, several tools have been developed to predict antibody tolerance. One possibility to estimate the potential immunogenicity of non-human antibodies is to calculate the level of identity, given by the Germinality Index (GI), which compares the corresponding framework regions with the most similar human germline encoding framework regions [36]. The GI indirectly predicts the tolerance of the corresponding variable regions, based on the assumption that germline encoding sequences are optimally tolerated, as they represent part of the IgM repertoire. Out of the three neutralizing scFv, ELC76 was the most human-like antibody fragment with a mean GI value of 91.2% (87.9% for VH and 94.4% for VL) followed by ELC18 with 87.8% (85.7% for VH and 89.9% for VL). For comparison, the average GI value of 500 scFv isolated from the human naïve antibody gene library HAL7/8 [60] was 95.7%. The high humanness of the antibodies isolated against BoNT/E3 predict a high tolerance and studies with the chimeric antibody lumiliximab, consisting of the variable regions of a macaque antibody in combination with the human constant regions, showed a good immune tolerance in human [61,62]. As an alternative, germline humanization of antibodies provides a potentially promising method for increasing immune tolerance for human treatment [63]. This method was successfully used for the humanization of 35PA_38_, a scFv neutralizing the anthrax lethal toxin, isolated from a macaque immune library and this increased its GI value to 97.8% [64]. For further development, germline humanization of the BoNT/E3 specific antibodies reported here would be a reasonable strategy. The antibody ELC18 is a promising candidate for future therapeutic development.

## Supporting Information

S1 File Raw Data of in vitro inhibiton ELISAs, ex vivo assays and in vivo neutralization. (ODS)
